# Geniposide suppressed OX-LDL-induced osteoblast apoptosis by regulating the NRF2/NF-κB signaling pathway

**DOI:** 10.1186/s13018-023-04125-5

**Published:** 2023-08-30

**Authors:** Yaosheng Xiao, Shanshan Zhang, Yongjun Ye, Jincai Chen, Youjia Xu

**Affiliations:** 1 Medical College of Soochow University, Suzhou, 215123 China; 2 Department of Orthopedics, First Affiliated Hospital of Gannan Medical University, Ganzhou, 341000 China; 3 College of Pharmacy, Gannan Medical University, Ganzhou, 341000 China; 4 Department of Orthopaedics, The Second Affiliated Hospital of Soochow University, Suzhou, 215004 China

**Keywords:** Osteoporosis, Geniposide, Osteoblast, Apoptosis, NRF2/NF-κB

## Abstract

**Background:**

Osteoporosis (OP), due to microarchitectural alterations, is associated with decreased bone mass, declined strength, and increased fracture risk. Increased osteoblast apoptosis contributes to the progression of OP. Natural compounds from herbs provide a rich resource for drug screening. Our previous investigation showed that geniposide (GEN), an effective compound from *Eucommia ulmoides*, could protect against the pathological development of OP induced by cholesterol accumulation.

**Methods:**

The rat OP models were duplicated. Dual-energy X-ray absorptiometry, hematoxylin and eosin staining, and immunohistochemistry were used to evaluate bone changes. TUNEL/DAPI staining assays were used for cell apoptosis detection. Protein expression was determined by western blotting assays.

**Results:**

A high-fat diet promoted OP development in vivo, and OX-LDL stimulated osteoblast apoptosis in vitro. GEN exhibited protective activities against OX-LDL-induced osteoblast apoptosis by increasing the NRF2 pathway and decreasing the NF-κB pathway. PDTC, an NF-κB inhibitor, could further promote the biological functions of GEN. In contrast, ML385, an NRF2 inhibitor, might eliminate GEN’s protection.

**Conclusion:**

GEN suppressed OX-LDL-induced osteoblast apoptosis by regulating the NRF2/NF-κB signaling pathway.

## Introduction

Osteoporosis (OP), a systemic skeletal disease, is characterized by bone microstructure destruction, increased risk of bone fragility and fracture, and high incidence rate [1]. Hyperlipidemia, referring to lipid metabolic abnormalities or disorders [2], can be featured as high cholesterol, high blood triglycerides, and mixed hyperlipidemia. It has been involved in the pathological development of many diseases, such as diabetes, atherosclerosis, and OP, posing a threat to people's health [3]. Low-density lipoprotein (LDL), a vehicle responsible for endogenous cholesterol, acts as the pathogenic substance in hyperlipidemia [4]. LDL has been considered a potential target for the prevention of cardiovascular diseases. Monitoring plasma LDL concentration becomes hyperlipidemia guidance for evaluating therapeutic effects. Oxidized LDL (OX-LDL) can be obtained through the enzymatic oxidation of LDL [5]. OX-LDL accumulation can be induced by oxidative stress, inflammation, and immune regulation. OX-LDL participates in bone regeneration processes. Hyperlipidemia in rats is related to decreased osteoblast number, reduced osteogenesis, and attenuated reconstruction of bone. Specifically, OX-LDL can reduce alkaline phosphatase (ALP) activity and induce osteoblast apoptosis [6].

Geniposide (GEN), one of the main bioactive constituents from the bark of *Eucommia ulmoides*, has been reported to treat OP. GEN has been reported to possess multiple pharmacological properties, including anti-inflammatory, anti-diabetes, neural protection, and rheumatoid arthritis protection [7]. In addition, GEN can reduce abnormal inflammatory responses and damage in rat bones, as indicated by decreased levels of TNF-α and IL-6 [8] and downregulated activity of the NF-κB pathway [9, 10]. Activation of the NF-κB pathway is associated with increased apoptosis in MC3T3-E1 cells [11]. Studies have shown that NRF2 is an important regulator of bone homeostasis [12], and its activation stimulates the endogenous antioxidant effect against the production of reactive oxygen species [13]. Our previous study shows that GEN can improve BMD and reverse the loss of bone trabecular in glucocorticoid-induced rat OP [14–16]. In addition, GEN enhances osteoblast differentiation and proliferation, ameliorates cholesterol accumulation, activates autophagy, and suppresses cell apoptosis in dexamethasone-treated MC3T3-E1 cells [14, 16]. Mechanically, the protective effects of GEN against OP might be associated with the inhibition of endoplasmic reticulum (ER) stress and activation of the GLP-1R signaling [14–16]. Therefore, GEN can be a potential agent for the treatment of OP. This article will investigate the inhibitory effects of GEN against OX-LDL-induced osteoblast apoptosis by regulating NF-κB and NRF2 signaling pathways.

## Materials and methods

### General information

According to the Declaration of Helsinki Principles, the protocol (GMU202011) was approved by the Institutional Animal Care and Use Committee of Gannan Medical University. The designed experiments were conducted in line with the principles for laboratory animal use and care in the European Community guidelines (EEC Directive of 1986; 86/609/EEC). Six-week-old (200 ± 20 g) male rats were included in an adaptive feeding in an SPF-grade room. The specific conditions with a cycle of 12-h lightness and darkness, 21–23 °C of temperature, and 45–55% of humidity were set. Food and water were freely accessible.

### Duplication of OP models in rats

All included rats were separated into four groups (6 rats/group) randomly. They are (1) the negative control (NC) group, treated with vehicle only; (2) the high fat (HF, obtained from Biotech, Beijing, China) group; (3) HF plus GEN (50 mg/kg/day by intragastrical administration; purchased from Shaanxi Jinkangtai Biotechnology Co., Ltd.; ≥ 98% of purity); and (4) HF plus GEN (100 mg/kg/day by intragastrical administration). Not any obvious untoward effects were recorded during the experiments. Rats were sacrificed after six months. The intact right femurs were collected and prepared for several evaluations, such as dual-energy X-ray absorptiometry, histochemical analysis, and immunohistochemical examination.

### Bone evaluation by dual-energy X-ray absorptiometry (DEXA)

Prepared femurs were located horizontally as instructed and evaluated by DEXA (Lunar Prodigy, GE Healthcare, Germany). The parameter BMD (g/cm^2^) in the femoral neck was measured after marking the region of interest (ROI) separately.

### Hematoxylin and eosin (HE) and immunohistochemistry (IHC) evaluation

Paraformaldehyde (4%, obtained from Beyotime, Shanghai, China) was used to fix the samples for 48 h, and EDTA solution (10%) was subsequently employed to decalcify for 20 days. Samples embedded in the paraffin were cut into 5-μm slices. After that, all slices were sequentially eluted in dimethyl benzene dewaxing for 30 min, ethanol (100%) for 6 min, ethanol (90%) for 3 min, ethanol (80%) for 2 min, distilled water, and PBS. HE (Solarbio Biotechnology, Beijing, China) was used for dyeing, and histologic analysis was performed.

Before IHC evaluation, slices were deparaffinized and washed with PBS. H_2_O_2_ (3%) was used to block the activity of endogenous peroxidase. Then, the goat serum (10%) was added and incubated for 30 min. The primary antibodies, such as Bcl-2 (1:200 dilution; Affinity Biosciences, Cincinnati, USA), cleaved-caspase3 (1:200 dilution; Affinity), p65 (1:200 dilution; Affinity), and NRF2 (1:200 dilution; Affinity) were added for co-incubation overnight at 4 °C. After washing with PBS, the horseradish peroxidase-conjugated secondary antibody (1:2000 dilution; Boster Biological Technology, Wuhan, China) was added for 30 min. All slices with immunostaining were further stained with DAB. Then, they were analyzed by ImageJ (an open-source image processing package).

### Cell culture

MC3T3-E1 cells were gained and grown in our previous study [14]. Simply, α-MEM was employed to culture MC3T3-E1 cells. Osteogenic differentiation was induced by treatment with an osteogenic induction medium (OIM) for 21 days. Cells were co-incubated overnight with OX-LDL (50 mM, Yiyuan Biotechnologies, Guangzhou, China) and GEN (20 μM and 50 μM, MedChemexpress, New Jersey, USA).

### CCK-8 assays

MC3T3-E1 cells (3000 cells/well) were seeded in 96-well plates. After the cells adhered, the culture medium in the wells was discarded. OX-LDL with 0 μg/mL, 25 μg/mL, 50 μg/mL, 100 μg/mL, and 200 μg/mL concentrations, respectively, was added for culture. Cell viability was measured after stimulation for 24 h. Specifically, the culture medium was removed, and 100 μL CCK-8 solution (Solarbio) was added in the darkness. Then, cells were incubated at 37 °C for 1 h. The wavelength of 450 nm was selected, and the optical density (OD) was measured using a microplate reader (Thermo Fisher Scientific Inc., Waltham, MA, USA).

### TUNEL/DAPI staining assays

24-well plates were used to culture cells (1 × 10^5^ cells/well). GEN with different concentrations was added for 24 h. Cells were sequentially washed with PBS, fixed with 4% paraformaldehyde for 30 min, and washed with PBS. After that, 50 μL immunostaining permeabilizing solution was added for 5 min at room temperature, and then 50 μL TUNEL detection solution (Beyotime) was added for 60 min in the darkness at room temperature. After washing with PBS, 4′,6-diamidino-2-phenylindole (DAPI, Solarbio) staining solution was added for 10 min at 37 °C in the darkness. The fluorescence microscope was used, and ImageJ was employed to analyze the fluorescent intensity.

### Western Blot

Total protein in MC3T3-E1 cells was extracted, and the concentrations of protein were detected by a BCA kit (Beyotime). 30 µg of each sample was subjected to 10–12% SDS-PAGE for separation, and the blots were then transferred onto PVDF membranes. After blocking with 5% skim milk in TBS for 1 h, the primary antibodies, including anti-Bcl-2 (1:1000 dilution; Affinity), anti-cleaved caspase 3 (1:1000 dilution; Cell Signaling Technology, Danvers, MA, USA), anti-p65 (1:1000 dilution; Affinity), anti-p-p65 (1:1000 dilution; Affinity), anti-NRF2 (1:1000 dilution; Affinity), anti-HO-1 (1:1000 dilution; Beyotime), and anti-β-actin (1:1000 dilution; Solarbio), were incubated with the membranes at 4 °C overnight. HRP-labeled goat anti-rabbit secondary antibodies (1:5000 dilution; Boster, Wuhan, China) were added at room temperature for 1 h. Finally, protein bands were measured by the enhanced chemiluminescence detection system and evaluated by ImageJ.

### Statistical analysis

GraphPad Prism 8 (GraphPad Software Inc., La Jolla, CA, USA) was used for statistical analysis. The statistical significance was analyzed by One-way analysis of variance (ANOVA) and Turkey’s post hoc test. *p* < 0.05 indicated a statistical difference.

## Results

### GEN ameliorated HF diet-induced OP in rat models

**Fig. 1:**
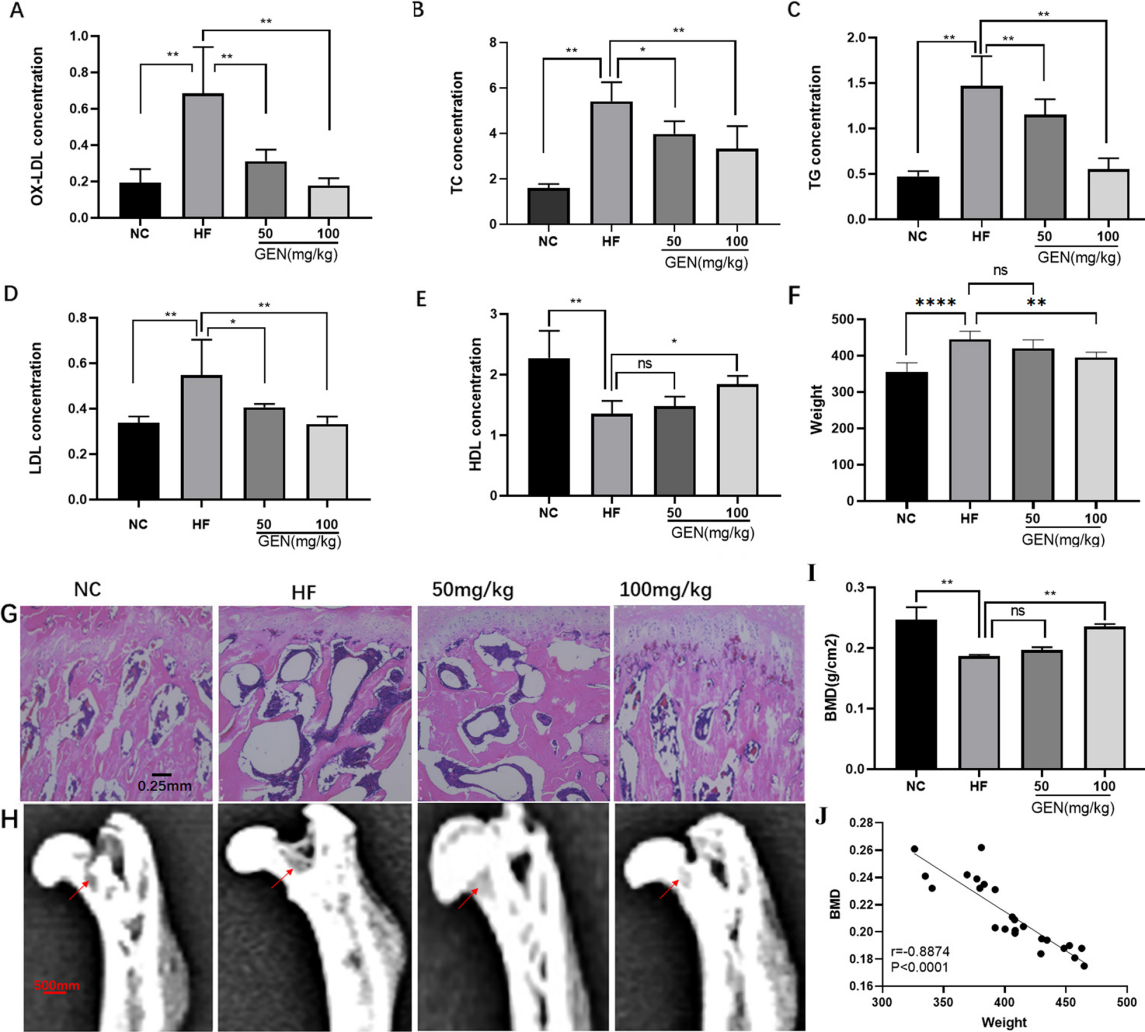
GEN ameliorated HF-induced OP in rat models (*n* = 6). **A**–**F** The serum levels of OX-LDL, TC, TG, LDL, HDL, and body weight were detected. (**G**) HE staining of the proximal femoral trabeculae was evaluated (40 × magnification). **H** The proximal femurs were scanned by DEXA. **I** The values of BMD in the femoral neck were analyzed. **J** The correlation between weight and BMD was analyzed. *p < 0.05, **P˂0.01. NC, negative control; HF, high fat; 50 mg/kg, HF + 50 mg/kg GEN; 100 mg/kg, HF + 100 mg/kg GEN

HF diet may induce damage to bone metabolism. Firstly, the HF diet in rats resulted in hyperlipidemia (Fig. 1A–F), as indicated by increased serum levels of OX-LDL, total cholesterol (TC), triglyceride (TG), LDL, and body weight, and decreased production of high-density lipoprotein (HDL). In HF-induced rat models, trabecular bone disorganization and thinning were observed by HE staining (Fig. 1G). Data from DEXA on the proximal femur showed that an HF diet could induce a loss of bone trabecula (Fig. 1H) and decrease the value of BMD (Fig. 1I). The changes in body weight were negatively correlated with BMD (Fig. 1J). Treatment with GEN (50 mg/kg and 100 mg/kg, respectively) could effectively reverse HF diet-induced alterations in serum OX-LDL, TC, TG, LDL, and HDL, ameliorate pathological changes in bone trabecula, and increase BMD values, protecting against HF diet-induced rat OP.

### GEN attenuated HF-induced osteoblast apoptosis

**Fig. 2:**
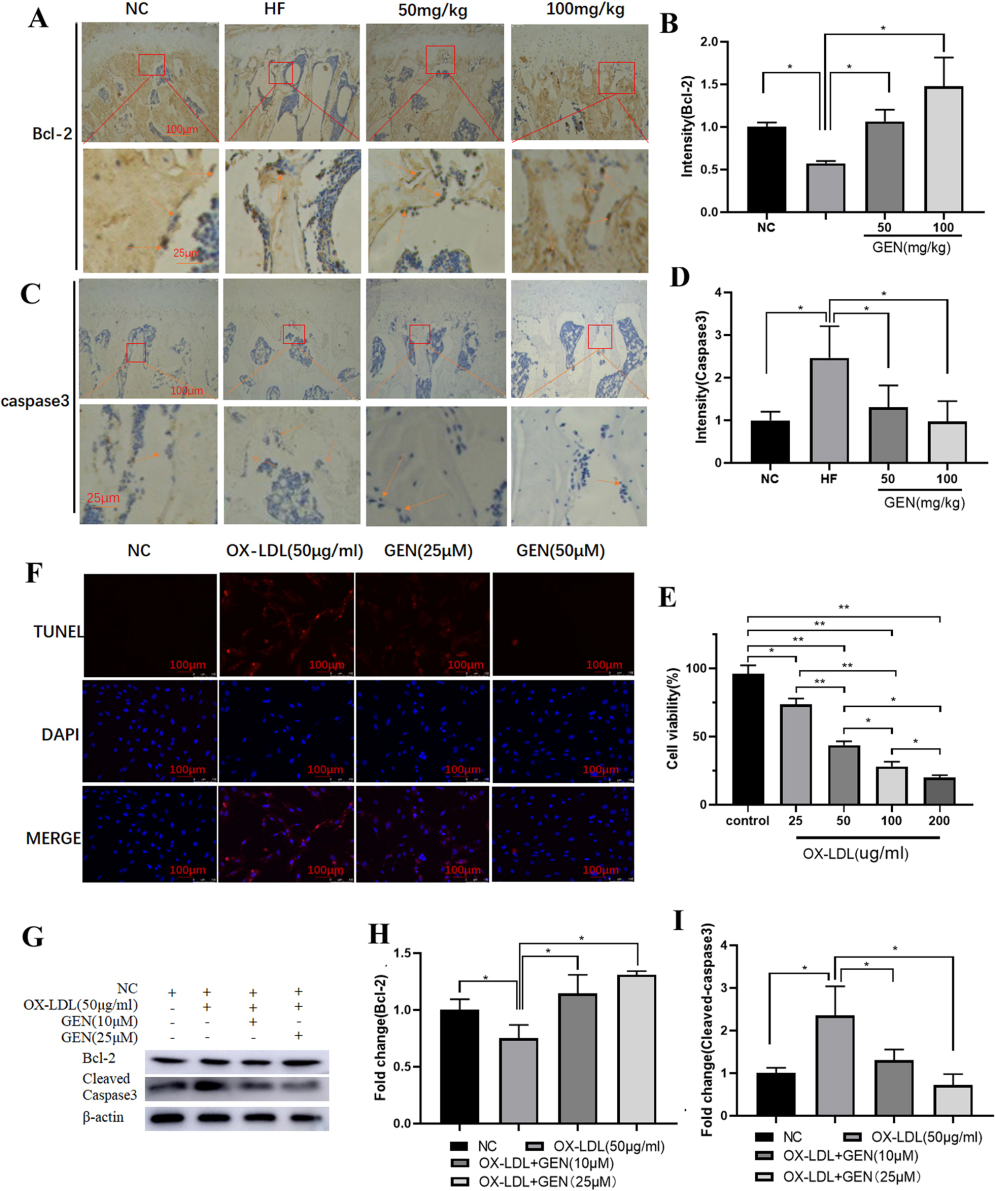
The inhibitory effects of GEN against HF-induced osteoblast apoptosis in vivo and in vitro. The changes of Bcl-2 (**A**, **B**) and caspase3 (**C, D**) in IHC (× 40 magnification) were analyzed. **E** CCK-8 assays were performed. **F** TUNEL staining assays were performed for detection of cell apoptosis. The protein expression of Bcl-2 (**G**, **H**) and cleaved-caspase3 **G**, **I** in OX-LDL-treated MC3T3-E1 cells was determined by western blot. *p < 0.05, **p˂0.01. NC, negative control; HF, high fat; 50 mg/kg, HF + 50 mg/kg GEN; 100 mg/kg, HF + 100 mg/kg GEN

To discover the underlying mechanisms of GEN in ameliorating HF diet-induced loss of bone trabecula, osteoblast apoptosis was detected. The in vivo IHC showed that the HF diet could decrease the expression of Bcl-2 and increase the expression of caspase3 in the bone trabecula of the proximal femurs (Fig. 2A-D). In vitro study, CCK-8 assays were performed to explore the effect of OX-LDL on cell viability. The results showed that OX-LDL could significantly trigger apoptosis in a dose-dependent manner (Fig. 2E), and the IC_50_ value was 57.2 μg/mL. OX-LDL at a dose of 50 μg/mL was chosen for the following experiments. TUNEL/DAPI staining assays showed that OX-LDL significantly increased osteoblast apoptosis (Fig. 2F). Consistently, the Bcl-2 protein expression (Fig. 2G, H) was down-regulated, and the cleaved-caspase3 protein expression (Fig. 2G, I) was up-regulated by OX-LDL in MC3T3-E1 cells. The doses of GEN (10 µM and 25 µM) were used in vitro study as those in our previous study [15, 17]. GEN could effectively attenuate HF-induced osteoblast apoptosis in vivo and in vitro. Collectively, the protective activity of GEN against HF diet-induced loss of bone trabecula might be associated with the inhibition of osteoblast apoptosis.

### GEN inhibited OX-LDL-induced osteoblast apoptosis by down regulating NF-κB pathway

**Fig. 3:**
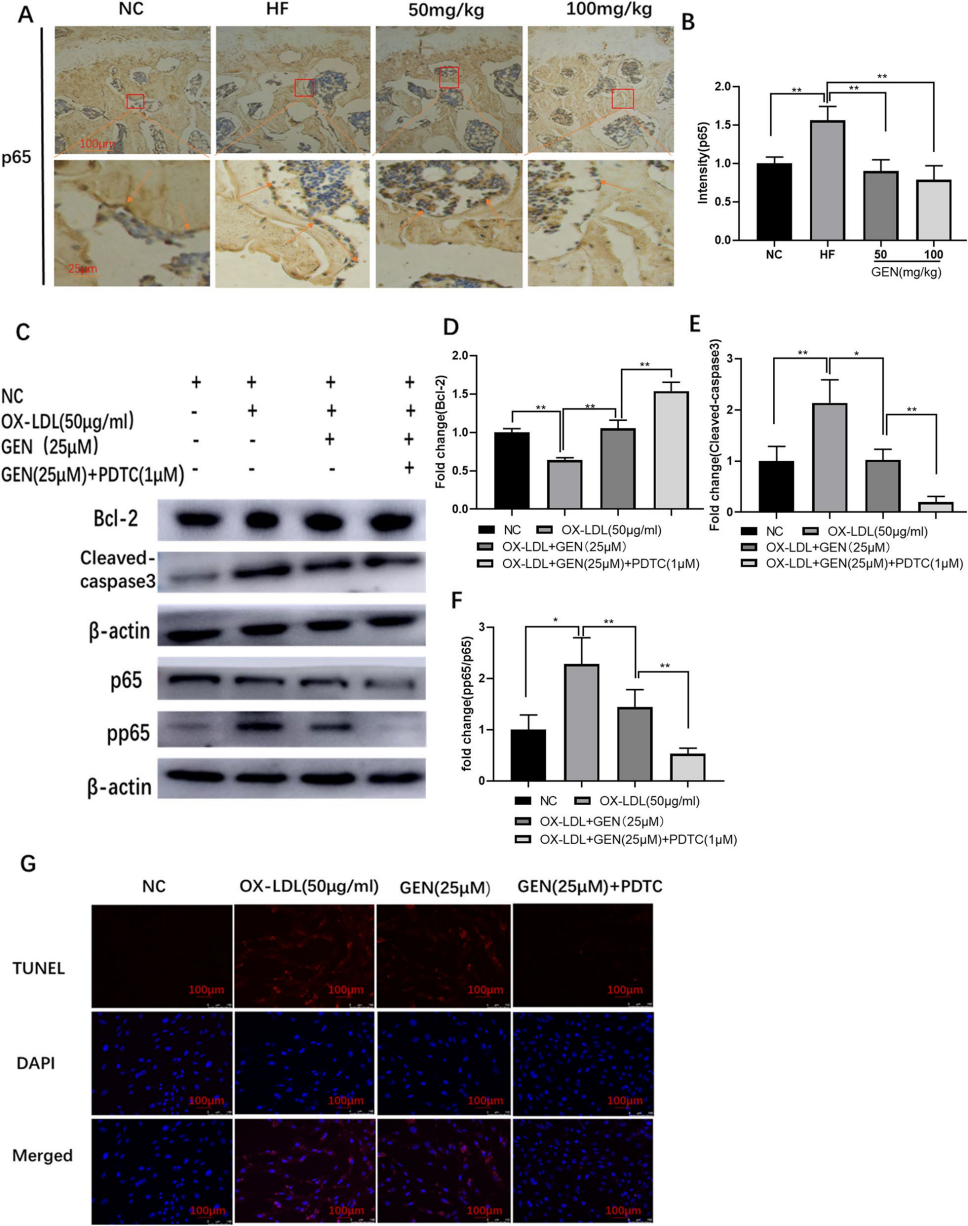
GEN inhibited OX-LDL-induced osteoblast apoptosis by down regulating NF-κB pathway. **A**, **B** The changes of p65 in IHC (× 40 magnification) were analyzed. The protein expression of Bcl-2 (**C**, **D**), cleaved-caspase3 (**C**, **E**), and p-p65/p65 **C**, **F** were detected by western blot in HF/PDTC-treated MC3T3-E1 cells. **G** TUNEL staining assays were performed for detection of cell apoptosis. *p < 0.05; **p < 0.01, NC, negative control; HF, high fat; 50 mg/kg, HF + 50 mg/kg GEN; 100 mg/kg, HF + 100 mg/kg GEN

To further explore the potential mechanism of GEN in inhibiting OX-LDL-induced osteoblast apoptosis, the NF-κB pathway was detected. As the results, in Fig. 3A, B, GEN could significantly prevent HF-induced p65 expression in vivo. Consistently, the expression of cleaved caspase3 and p-p65/p65 was down-regulated, and the expression of Bcl-2 was up-regulated by GEN in OX-LDL-treated MC3T3-E1 cells (Fig. 3C–F). Furthermore, combined treatment with the NF-κB antagonist PDTC (1 µM), the inhibitory effects of GEN against OX-LDL-induced osteoblast apoptosis (Fig. 3G) were further enhanced. These indicated that GEN might synergy with PDTC to inhibit OX-LDL-induced osteoblast apoptosis.

### GEN inhibited OX-LDL-induced osteoblast apoptosis by up regulating NRF2 pathway

**Fig. 4:**
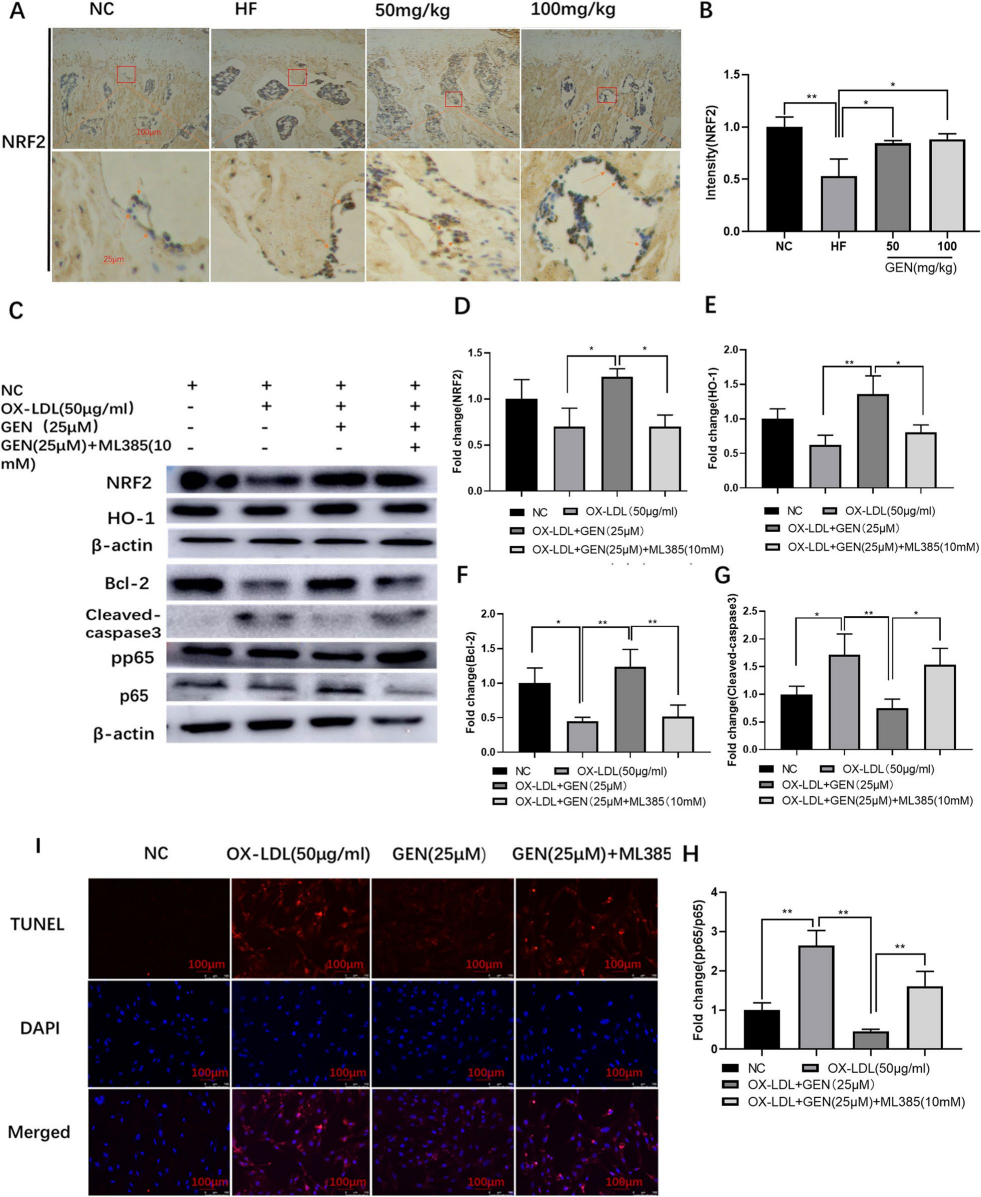
GEN inhibited OX-LDL-induced osteoblast apoptosis by up regulating NRF2 pathway. **A**, **B** The changes of NRF2 in IHC (× 40 magnification) were analyzed. The protein expression of NRF2 (**C**, **D**), HO-1 (**C**, **E**), Bcl-2 (**C**, **F**), cleaved-caspase3 (**C, G**), p-p65/p65 (**C, H**) were detected by western blot in OX-LDL/ML385-treated MC3T3-E1 cells. **I** TUNEL staining assays were performed for detection of cell apoptosis. *p < 0.05; **p < 0.01, NC, negative control; HF, high fat; 50 mg/kg, HF + 50 mg/kg GEN; 100 mg/kg, HF + 100 mg/kg GEN

We found that the HF-reduced expression of NRF2 in vivo was down-regulated by GEN in IHC assays (Fig. 4A, B). In OX-LDL-treated MC3T3-E1 cells, the protein expression of NRF2 and HO-1 was down-regulated (Fig. 4C–E). To further explore the roles of NRF2 in GEN-mediated protection against OX-LDL-induced osteoblast apoptosis, the NRF2 antagonist ML385 (10 mM) was added. As expected, ML385 could down-regulate Bcl-2 expression (Fig. 4C, F), up-regulate cleaved-caspase3 (Fig. 4C, G) and p-p65/p65 expression (Fig. 4C, H), and enhance apoptosis ratio (Fig. 4I), abolishing the protective effects of GEN. Collectively, GEN suppressed OX-LDL-induced osteoblast apoptosisby up-regulating the NRF2 pathway.

## Discussion

OP is characterized by decreased strength and microarchitectural alterations in bone tissues, increasing the fracture risk. A higher prevalence and incidence of OP-related bone fracture are observed in females than those in males. However, male rats were employed in this study by avoiding the disturbance of estrogen, which has been reported to be negatively associated with OP development [18]. Dysregulated bone metabolism contributes to the incidence of OP pathological development. Lipid maintains the homeostasis of bone metabolism. However, dysregulation of lipid metabolism, particularly oxidized lipids, has been recently reported to participate in the development of many diseases, including atherosclerosis and OP [19]. Clinical data have been analyzed that dyslipidemia is negatively correlated with BMD, and the potential mechanisms might be associated with increased oxidative stress and systemic inflammation [20]. In this article, we consistently found that OX-LDL could increase osteoblast apoptosis and promote OP development, which was associated with increased activity of the NF-κB pathway and decreased activity of the NRF2 pathway. Interestingly, GEN, a bioactive iridoid isolated from *E. ulmoides*, exhibited protective effects against OX-LDL-induced osteoblast apoptosis by mediating NF-κB and NRF2 pathways, protecting OP development.

Hyperlipidemia indicates abnormally elevated levels of lipids or lipoproteins in the blood [21], and it is often associated with dietary disorders, obesity, and genetic diseases, such as familial hypercholesterolemia [22]. Hyperlipidemia is characterized by high levels of TC, TG, and LDL-C in the blood [23]. OX-LDL can be produced from LDL due to oxidative stress. OX-LDL has become an important indicator of hyperlipidemia [24] and is reported to decrease bone metabolic rate [25]. Our study found that the HF diet consistently induced high levels of TC, TG, LDL, and OX-LDL and low levels of HDL, indicating the successful establishment of hyperlipidemia. In addition, the HF diet also decreased the values of BMD and promoted the development of OP in rat models.

HF diet-associated hyperlipidemia may lead to lipid accumulation, structural and functional abnormalities, and aberrant stimulation of inflammatory responses. Several studies have reported that an HF diet is closely associated with inflammation [26, 27]. NF-κB, one of the members in the family of transcription factors, often functions in most cells [28] and involves in the immune and inflammatory responses and affecting cell differentiation, apoptosis, and tissue damage [29]. It has been shown that an HF diet may induce high levels of IL-1β, IL-6, and TNFα by up-regulating the expression of TLR4 and NF-κB pathways [30]. HF diet also triggers glucose intolerance and insulin resistance, which might be associated with the up-regulation of NF-κB-mediated inflammation [31]. Recently, it has been reported that an HF diet can increase osteoblast apoptosis and promote OP development [32]. It has been shown that activation of the NF-κB pathway can increase MC3T3-E1 cell apoptosis [33]. In addition, PDTC, an NF-κB inhibitor, can reduce the number of apoptotic cells [34]. Consistently, we found that the HF diet increased the expression of caspase-3 and p65 and decreased the expression of Bcl-2 in rats. These suggested a relationship between OP development and HF diet-promoted osteoblast apoptosis and -activated NF-κB pathway. Further, we also found that blocking the NF-κB pathway by PDTC could ameliorate OX-LDL-induced osteoblast apoptosis.

HF diet-associated hyperlipidemia is also associated with oxidative stress. It has been shown that an HF diet may down-regulate NRF2 expression [35]. In hepatocytes, an HF diet can down-regulate the expression of the SIRT6/PGC-1α/ENDOG signaling pathway, inducing oxidative stress [36]. Recently, NRF2 has been regarded as a transcription factor for bone balance, participating in the regulation of OP development [37]. Sham-operated or OVX *NRF2*^−/−^ mice have reported a decreased value of BMD [38]. *NRF2* deficiency tends to down-regulate osteoblast marker expression and increase osteoclast marker levels in sham-operated animals, suggesting it can enhance bone turnover [39, 40]. In addition, it is reported that the expression of NRF2 was decreased in the bone of osteoporotic rats [41]. Thus, NRF2 may serve as a therapeutic target to maintain bone mass. Consistently, we found that the expression of NRF2 was down-regulated by OX-LDL, and NRF2 inhibitor ML385 could potentiate OX-LDL-mediated osteoblast apoptosis.

GEN, a bioactive iridoid, has many pharmacological effects, such as anti-inflammation, anti-angiogenic detoxification, anti-oxidation, and immunomodulation [42]. GEN can restore the abnormally elevated levels of TNF-α and IL-6 in rats, inhibit B cell proliferation and autoantibody production, and prevent the function of effector T cells, achieving to reduce local inflammation [43]. GEN may inhibit osteoblast apoptosis through NF-κB and NRF2 signaling pathways [44]. Our previous study shows that GEN ameliorates dexamethasone (DEX)-induced cholesterol accumulation and endoplasmic reticulum (ER) stress by up-regulating the expression of GLP-1R. In addition, GEN also exhibits inhibitory effects against DEX-induced osteoblast apoptosis by stimulating autophagy. In this study, we further investigated that GEN exhibited protective effects against HF diet-induced rat OP by inhibiting osteoblast apoptosis. The possible molecular mechanism of GEN in protecting against OX-LDL-induced osteoblast apoptosis might be associated with the up-regulation of the NRF2 pathway and down-regulation of the NF-κB pathway.

Many anti-inflammatory drugs can inhibit the activation of the NF-κB pathway, while anti-oxidant drugs usually perform the cytoprotective function by activating the NRF2 pathway [45]. It is of concern that many drugs have dual anti-inflammatory and anti-oxidant functions [46]. Two pathways can often accept the same chemical signal but respond differently. Both NF-κB and NRF2 can respond to oxidative stress stimuli and play a role in the regulation of redox [47]. It remains unclear whether this response and modulation to co-stimulation are accomplished separately by the two pathways or whether there are some synergistic or antagonistic mechanisms. Unfortunately, our study did not explore the specific relationship between NRF2 and NF-κB and the principles of interaction, and no deeper exploration has been made into how the respective pathways function. More efforts are still needed in the exploration of the pharmacological activity of GEN in displaying dual functions anti-inflammation and anti-oxidation.

Some limitations of this study are concerned. For example, the animal diet in the negative control group was not refined. The basal diet is obtained commercially, and it might have phytochemical contaminations, which could affect the results of the current study. GEN may inhibit the proliferation of osteoclasts [48]. However, the potential mechanisms remain unclear. Genipin, the geniposide aglycon, has been reported to inhibit RANKL-induced osteoclast differentiation by suppressing c-Fos protein proteolysis and IκB degradation [49]. However, the effects of GEN on osteoclastogenesis and osteoclast activities are still unclear, and no such studies have been reported. Further studies are still needed.

## Conclusion

To sum up, an HF diet could decrease BMD values and promote OP development in rats. OX-LDL induced osteoblast apoptosis by upregulating the NF-κB pathway and downregulating the NRF2 pathway in vitro. GEN exhibited protective effects against OX-LDL-induced osteoblast apoptosis by mediating NF-κB and NRF2 signaling pathways.

## Data Availability

All data generated or analyzed during this study are included in this published article.
